# Good Syndrome Presenting With Pulmonary Abnormalities Four Years After Thymectomy

**DOI:** 10.1002/rcr2.70683

**Published:** 2026-07-13

**Authors:** Tomohide Hanawa, Tomohiro Akaba, Yuka Kaizu, Miki Kubo, Ayaka Fukuda, Mayu Fujii, Mamiko Muramatsu, Etsuko Tagaya

**Affiliations:** ^1^ Department of Respiratory Medicine Tokyo Women's Medical University Tokyo Japan; ^2^ Department of Pulmonary Medicine Tokyo Metropolitan Okubo Hospital Tokyo Japan; ^3^ Department of Radiation Oncology Tokyo Women's Medical University Tokyo Japan

**Keywords:** good syndrome, hypogammaglobulinemia, recurrent infection, thymoma

## Abstract

Good syndrome is a rare immunodeficiency associated with thymoma, characterized by hypogammaglobulinemia and marked B‐cell depletion. A 72‐year‐old woman who had undergone thymectomy 4 years earlier was referred to our hospital due to recurrent respiratory infections. Chest computed tomography revealed patchy ground‐glass opacities without evidence of thymoma recurrence. Bronchoalveolar lavage showed lymphocytosis, suggesting that the pulmonary abnormalities had possible inflammatory features, although infection could not be fully excluded. A short course of corticosteroids was added to empiric antibiotic therapy with careful monitoring, and the respiratory symptoms and radiological abnormalities improved after treatment. Because of her history of thymoma, further immunologic evaluation was performed and revealed profound hypogammaglobulinemia with near absence of peripheral B cells, leading to a diagnosis of Good syndrome. Intravenous immunoglobulin therapy was initiated, resulting in an increase in serum IgG levels and prevention of further respiratory infections. This case highlights that Good syndrome may become clinically apparent years after thymectomy and should be considered in patients with recurrent respiratory infections and unexplained pulmonary abnormalities.

## Graphical abstract

Good syndrome may become clinically apparent several years after thymectomy and can present with pulmonary abnormalities. In this case, ground‐glass opacities and BAL lymphocytosis suggested possible inflammatory features, although infection could not be excluded. Subsequent immunologic evaluation revealed hypogammaglobulinemia, leading to the diagnosis of Good syndrome and initiation of intravenous immunoglobulin replacement therapy.

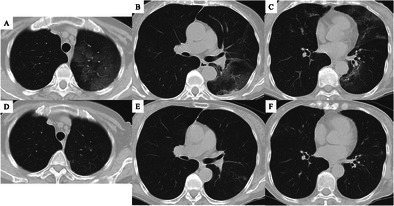

## 1 Introduction

Good syndrome is a rare adult‐onset immunodeficiency associated with thymoma and characterized by hypogammaglobulinemia, marked B‐cell depletion, and variable T‐cell dysfunction, leading to recurrent opportunistic infections [1, 2]. We report a patient who developed recurrent respiratory infections and pulmonary abnormalities 4 years after complete thymoma resection. The long interval after surgery and the presence of pulmonary opacities made the diagnosis challenging.

## 2 Case Report

A 72‐year‐old woman was referred to our hospital for persistent and relapsing respiratory symptoms. About 2 months before admission, she developed repeated upper and lower respiratory tract infections. She was treated at a local clinic with antibiotics, but symptoms improved only temporarily. Because fever and cough recurred despite antibiotic therapy, she was referred for further evaluation.

She had undergone complete resection of a type B1 thymoma 4 years earlier. The postoperative course had been uneventful. There was no tumour recurrence and no prior history of recurrent infections. She had no smoking history or allergies. There was no history of newly initiated medications. On admission, vital signs were stable except for temperature (37.3°C): blood pressure 129/75 mmHg, pulse 77/min, respiratory rate 18/min, and SpO_2_ 98% on room air. Physical examination was unremarkable. Chest computed tomography showed patchy ground‐glass opacities with interlobular septal thickening in the left lung (Figure 1A–C). There was no evidence of thymoma recurrence. Laboratory tests showed a white blood cell count of 5100/μL, an absolute neutrophil count of 4396/μL, an absolute lymphocyte count of 351/μL, and a C‐reactive protein level of 2.90 mg/dL. Serum levels of KL‐6, β‐D‐glucan, and soluble IL‐2 receptor were 289 U/mL, ≤ 5.0 pg/mL, and 785 U/mL, respectively. Rheumatoid factor, antinuclear antibody, MPO‐ANCA, and PR3‐ANCA were negative. *Trichosporon asahii* antibody and T‐SPOT were negative. Multiplex PCR using a nasal swab sample (FilmArray Respiratory Panel) was also negative for common respiratory viruses and atypical bacteria.

**FIGURE 1:**
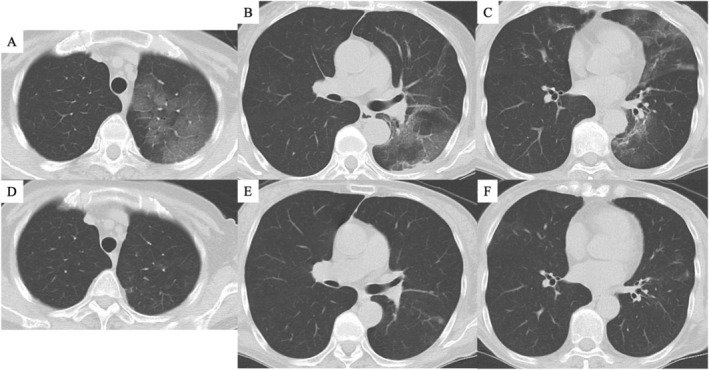
Chest CT findings. (A–C) On admission, patchy ground‐glass opacities with interlobular septal thickening are seen in the left lung. (D–F) After treatment, the opacities markedly resolved.

Empiric ceftriaxone therapy was initiated on hospital Day 1. Flexible bronchoscopy was performed on hospital Day 2 to evaluate the cause of the pulmonary opacities. Bronchoalveolar lavage (BAL) fluid analysis showed lymphocytosis (lymphocytes 65.9%) with a CD4/CD8 ratio of 0.31. BAL culture yielded a small number of *Haemophilus influenzae* with otherwise normal flora. *Pneumocystis jirovecii* PCR using BAL fluid was negative. Differential diagnoses included interstitial pneumonia, viral pneumonia, and atypical bacterial pneumonia. Because imaging findings and BAL lymphocytosis suggested possible inflammatory features, oral prednisolone (0.5 mg/kg/day) was added to empiric ceftriaxone therapy on hospital Day 2 with close monitoring. Fever resolved and respiratory symptoms improved shortly after treatment.

In parallel with the bronchoscopic evaluation, we assessed her immune status given her history of thymoma. Serum immunoglobulin levels were found to be markedly reduced: IgG 260 mg/dL (reference 870–1700), IgA 5 mg/dL (110–410), and IgM < 5 mg/dL (33–190). Flow cytometry showed near absence of CD19‐positive B cells and reduced CD4‐positive T cells. These findings, together with the history of recurrent infections, established the diagnosis of Good syndrome. Intravenous immunoglobulin (IVIg) was administered at a dose of 10 g on hospital days 7 and 8. Because the serum IgG level subsequently decreased, an additional 10‐g dose was administered on hospital Day 18. Monthly IVIg replacement therapy was continued thereafter. Serum IgG increased to > 800 mg/dL, and respiratory symptoms resolved completely. Prednisolone was tapered and discontinued within 10 weeks without relapse. Follow‐up CT approximately 3 weeks after admission showed resolution of the ground‐glass opacities (Figure 1D–F). She remained stable on monthly IVIg replacement therapy and had no further infectious episodes during 2 years of follow‐up after initiation of IVIg. The clinical course is summarized in Figure 2.

**FIGURE 2:**
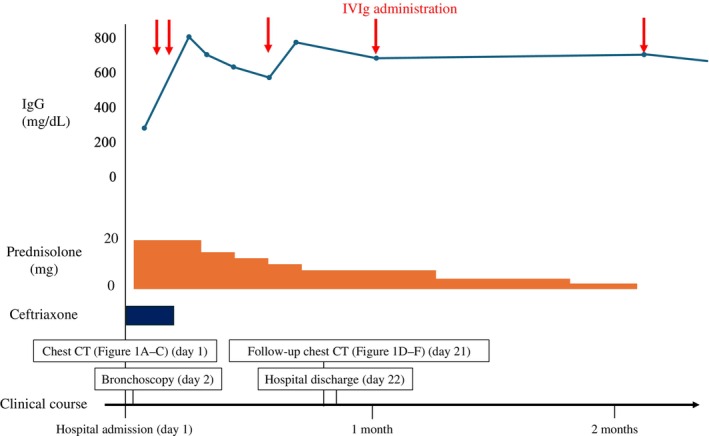
Clinical course. The timing of antimicrobial therapy, bronchoscopy, corticosteroid therapy, IVIg administration, follow‐up CT, and serum IgG measurements is shown. Serum IgG values were measured immediately before each IVIg administration and are presented as pre‐dose levels. The patient remained free from further infections during 2 years of follow‐up after initiation of IVIg.

## 3 Discussion

This case highlights that Good syndrome may become clinically apparent several years after thymectomy and can present with pulmonary abnormalities. In the present case, ground‐glass opacities on CT and BAL lymphocytosis suggested possible inflammatory features, although infection could not be fully excluded. Recognition of the prior thymoma prompted immunologic testing and led to the correct diagnosis.

Good syndrome is characterized by thymoma‐associated immunodeficiency with hypogammaglobulinemia and reduced or absent B cells. Hypogammaglobulinemia and reduced B‐cell numbers can also be seen in common variable immunodeficiency (CVID); however, in the present case, the history of thymoma supported the diagnosis of Good syndrome rather than CVID. T‐cell abnormalities, including reduced CD4‐positive cells, may further increase susceptibility to infection. Detection of *H. influenzae* in BAL fluid may have reflected impaired bacterial clearance, bacterial infection, or airway colonization in the setting of impaired humoral immunity [1, 3]. Therefore, a bacterial contribution could not be fully excluded. BAL lymphocytosis and improvement after treatment suggested that inflammatory features may have contributed to the pulmonary abnormalities, but the improvement cannot be attributed specifically to corticosteroid therapy alone. Viral infection could not be fully excluded, although multiplex PCR using a nasal swab sample did not identify a causative pathogen. Although corticosteroids may increase infection risk, short‐term use may be considered with careful monitoring when pulmonary abnormalities with possible inflammatory features are suspected.

Only limited cases have described pulmonary abnormalities with possible inflammatory features as the initial presentation of Good syndrome. However, various pulmonary manifestations, including interstitial lung abnormalities, have been reported in Good syndrome [4, 5]. In CVID, granulomatous‐lymphocytic interstitial lung disease (GLILD) is a recognized pulmonary manifestation characterized by lymphocytic infiltration and granulomatous inflammation. Although GLILD is most commonly described in CVID, GLILD‐like involvement has occasionally been reported in Good syndrome [4]. In the present case, ground‐glass opacities, BAL lymphocytosis, and improvement after treatment suggested possible inflammatory pulmonary involvement. However, pathological confirmation was not obtained, infection was not completely excluded, and radiological findings were not typical of GLILD. Therefore, the pulmonary abnormalities should be interpreted cautiously as non‐specific pulmonary involvement with possible inflammatory features. Nevertheless, IVIg replacement therapy remains the mainstay of treatment. IgG trough levels above 600–800 mg/dL are generally recommended, with dosing individualized according to clinical course.

A limitation is that immunoglobulin levels were not assessed before or after thymectomy. Immune evaluation was performed only after recurrent infections developed. Another limitation is that the aetiology of the pulmonary abnormalities could not be determined with certainty because pathological confirmation was not obtained and infection could not be completely excluded. Because thymectomy does not correct the underlying immunodeficiency, earlier and periodic immunologic assessment should be considered in patients with thymoma.

In conclusion, Good syndrome should be considered in patients with a history of thymoma who develop unexplained recurrent respiratory infections or pulmonary abnormalities, even years after thymectomy. Careful immunologic assessment and long‐term monitoring are essential.

## Author Contributions

**Tomohide Hanawa:** drafting the manuscript. **Tomohiro Akaba:** supervision and revision of the manuscript. **Yuka Kaizu:** revision of the manuscript. **Miki Kubo:** revision of the manuscript. **Ayaka Fukuda:** revision of the manuscript. **Mayu Fujii:** revision of the manuscript. **Mamiko Muramatsu:** revision of the manuscript. **Etsuko Tagaya:** supervision and final approval of the manuscript.

## Consent

The authors declare that written informed consent was obtained for the publication of this manuscript and accompanying images using the consent form provided by the Journal.

## Conflicts of Interest

The authors declare no conflicts of interest.

## Data Availability

The data that support the findings of this study are available from the corresponding author upon reasonable request.

## References

[1] P. Kelleher and S. A. Misbah , “What Is Good's Syndrome? Immunological Abnormalities in Patients With Thymoma,” Journal of Clinical Pathology 56, no. 1 (2003): 12–16. 12499426 10.1136/jcp.56.1.12 PMC1769851

[2] M. Malphettes , L. Gérard , L. Galicier , et al., “Good Syndrome: An Adult‐Onset Immunodeficiency Remarkable for Its High Incidence of Invasive Infections and Autoimmune Complications,” Clinical Infectious Diseases 61, no. 2 (2015): e13–e19. 25828999 10.1093/cid/civ269

[3] T. Kelesidis and O. Yang , “Good's Syndrome Remains a Mystery After 55 Years: A Systematic Review of the Scientific Evidence,” Clinical Immunology 135, no. 3 (2010): 347–363. 20149753 10.1016/j.clim.2010.01.006 PMC8071094

[4] K. Gocho , T. Tanaka , K. Furuhashi , et al., “Granulomatous‐Lymphocytic Interstitial Lung Disease Associated With Good's Syndrome That Responded to Immunoglobulin Therapy,” Internal Medicine 60, no. 19 (2021): 3137–3142. 33716283 10.2169/internalmedicine.6456-20 PMC8545647

[5] S. Agarwal and C. Cunningham‐Rundles , “Thymoma and Immunodeficiency (Good Syndrome): A Report of 2 Unusual Cases and Review of the Literature,” Annals of Allergy, Asthma & Immunology 98, no. 2 (2007): 185–190. 10.1016/S1081-1206(10)60695-3 PMC3102047 17304889

